# Does Natural Killer Cell Deficiency (NKD) Increase the Risk of Cancer? NKD May Increase the Risk of Some Virus Induced Cancer

**DOI:** 10.3389/fimmu.2019.01703

**Published:** 2019-07-19

**Authors:** Won Young Moon, Simon J. Powis

**Affiliations:** ^1^Taunton and Somerset NHS Foundation Trust, Taunton, United Kingdom; ^2^School of Medicine and Biological Sciences Research Complex, University of St. Andrews, St. Andrews, Scotland

**Keywords:** NKD, NK cells, oncogenic viral infections, CD56^bright^ NK cells, MDS-AML

## Abstract

Natural killer cell deficiency (NKD) is a primary immunodeficiency where the main defect lies in CD56^+^CD3^−^ natural killer (NK) cells which mediate cytotoxicity against tumors. Most cases are observed in children and adolescents with recurrent viral infections and cancer. *GATA2* and *MCM4* mutations are found in NKD patients with cancer. However, the question remains unclear whether NKD increases the risk of cancer. Mutations in the second zinc finger of *GATA2* cause both NKD and haematopoietic malignancies. *MCM4* splice site mutations are found in NKD patients and they increase susceptibility to DNA instability during replication. *IRF8, RTEL1*, and *FCGR3A* mutations are associated with NKD but their associations with cancer are unknown. Based on the studies, it is hypothesized that genetic mutations alone are sufficient to cause cancer. However, a number of NKD patients developed oncogenic viral infections which progressed into cancer. Here, we review the evidence of genetic mutations responsible for both NKD and cancer to identify whether NKD contributes to development of cancer. The findings provide insights into the role of NK cells in the prevention of cancer and the significance of assessing NK cell functions in susceptible individuals.

## Introduction

NK cells mediate cytotoxicity against tumor and viral infected cells. NK cells have activating and inhibitory receptors (1) which allow NK cells to selectively target pathogenic cells while preventing autoimmunity against autologous cell (2). The inhibitory receptors are activated by MHC-I while activating receptors are activated by activating ligands on pathogenic cells (2). NK cells play important role in cancer immunity as they mediate cytotoxicity against tumor cells that evade CD8^+^ T cells by up-regulating activating ligands and failing to express MHC-I (3). In addition, NK cells interact with dendritic cells and T cells to mediate cytotoxicity against tumor cells. Dendritic cells secrete cytokines which activate NK cells, resulting in NK cell secretion of perforin and expression of TNF ligands such as FASL and TRAIL that are responsible for tumor cell apoptosis (4). Conversely, NK cells present antigen debris that activate dendritic cells and they secrete IFNγ that activate T cells, resulting in cytotoxicity against tumor cells (4).

The role of NK cells in cancer immunity has been demonstrated from NK cell deficient mice and humans. NK1.1^+^CD3^−^ cell deficient transgenic mice showed poor tumor cell removal and increased metastasis compared to wild type *in vivo* (5). NK cells suppress spontaneous and MCA carcinogen induced tumor formation in mice (3). Furthermore, cancer patients with low peripheral or intratumoral NK cell activity have poorer survival rates (6) and low NK cell cytotoxicity facilitates the progression of precancerous disease into cancer (7) as well as metastases (8). An 11-year cohort study of Japanese residents illustrated that participants in the lowest tertile of natural cytotoxicity were more likely to develop cancer (9).

In humans, NKD has been defined as a primary defect in the number or function of CD56^+^CD3^−^ NK cell in peripheral blood which is caused by genetic abnormalities that persist over time (10). NKD can be subdivided into classical natural killer cell deficiency (CNKD) and functional natural killer cell deficiency (FNKD) (10). CNKD is defect in both number and function of NK cells in peripheral blood whereas FNKD results in only a functional defect (10). The mutations in *GATA2, MCM4, IRF8*, and *RTEL1* are associated with CNKD and substitutions in *FCGR3A* with FNKD (11).

The observational studies of NK cell deficient mice and humans have illuminated that NK cells contribute to tumor surveillance. The cancers have been observed in NKD patients with *GATA2* and *MCM4* mutations although not in patients with *IRF8, RTEL1*, and *FCGR3A* mutations (11). Despite these aspects, it is unclear whether NKD is a risk factor of cancer due to a difficulty in isolating NK cell defects as NK cells share receptors and cytokines with other immune cells. This poses the question of whether genetically defined NKDs weaken cancer immunity which cannot be overcome by other immune cells. If NKD increases susceptibility to at least some cancers, it would be worth assessing the number and cytotoxicity of NK cells to prevent such cancers. This review explores whether NKD increases the risk of cancer, based on existing literature.

## *GATA2* Mutations are Associated With AML-MDS

*GATA2* regulates the proliferation and survival of multi-potent haematopoietic stem cells (12). *GATA2* mutations selectively affect NK cells, B cells, dendritic cells, and monocytes (12). *GATA2* mutations have been observed in CNKD patients with haematopoietic malignancies, especially myelodysplastic syndrome, and acute myeloid leukemia (MDS-AML) (12). DNA sequencing of four MDS-AML families identified missense and frameshift mutations at Thr354 and Thr355 in the second zinc finger of *GATA2* (13). Both mutations resulted in the production of abnormal *GATA2* proteins that lacked an affinity to the consensus DNA and PU.1 transcription factor (13). When the mutant *GATA2* was transfected to promyelocytes, they proliferated substantially and failed to undergo apoptosis when treated with all-trans retinoic acid (ATRA) *in vitro* unlike those transfected with wild-type *GATA2* (14). The same *GATA2* mutations have been identified in Emberger syndrome (14), MonoMac syndrome (15) and dendritic cell, monocyte, B and NK lymphoid (DCML) deficiency (16), which accompany both MDS-AML and deficiency in NK cells. The observations reinforced that *GATA2* controls haematopoiesis and therefore its disruption results in cancer in affected cell lineages.

Mace et al. found that patients with *GATA2* mutations had CD56^bright^ subset loss and consequently increased proportion of CD56^dim^ subset, regardless of total NK cell count (12). In addition, there was significant downregulation of NKG2D and reduced cytotoxicity per NK cell in these patients, indicating that *GATA2* may be crucial for producing functional NK cells as well as maintaining CD56^bright^ NK cells (12). The downregulation of NKG2D compromises NK cell cytotoxicity in MDS and facilitates progression to AML (17). Furthermore, leukemic cells shed activating ligands to evade NK cell cytotoxicity, indicating role of NK cells in leukemia (18). Therefore, NKD caused by *GATA2* mutations is associated with increased risk of MDS-AML.

## *MCM4* Mutation Increases the Risk of Cancer

The oncogenic mechanism of *MCM4* mutation was revealed by the genetic mapping of mice erythrocytes, which showed that mutant *MCM4*^*Chaos*3^ allele causes T to A substitution in the downstream of the *MCM4* zinc finger, resulting in amino acid change in F345I (19). When mouse embryonic fibroblasts (MEFs) were transfected with DNAs containing *MCM4*^*Chaos*3^ allele, they were more susceptible to breakage when their DNAs were treated with aphidicolin. This suggested that *MCM4* mutations produce non-functional *MCM4* protein that has increased DNA instability upon replication stress.

Similarly, the genetic mapping of *MCM4* mutation of the four Irish children with CNKD identified premature stop codon, resulting in shortened *MCM4* proteins that lacked N-terminal domain containing F345I as observed in mice (20, 21). The affected children had reduced number of CD56^dim^ NK cell as well as reduced total number of NK cells and they had immature CD56^bright^ NK cells (12). One of them had lymphoproliferative disorder which progressed to EBV induced lymphoma (20). When the fibroblasts of the patients were stimulated with cytokines produced by CD56^bright^ NK cells, there were defects in their DNA replication (12). Therefore, high chromosomal breakage may have contributed to development of EBV induced lymphoma in this patient. The mutant alleles may not always lead to cancer even if they increase DNA instability as the complex interplay of genes and environmental factors determines occurrence of cancer (3). This explains why three out of four Irish CNKD children (20) did not develop cancer despite having the same *MCM4* mutations (22). Therefore, *MCM4* mutations are associated with both NKD and increased susceptibility to DNA instability upon stress.

## NKD Increases Susceptibility to Virus Related Cancer

**Figure 1:**
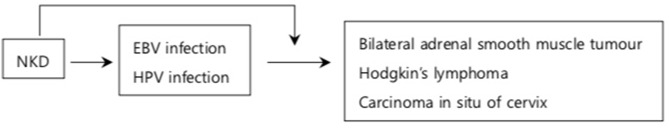
NKD and viral induced cancer. NKD results in reduced number of NK cells (11) and downregulation of activating receptors against oncogenic viruses (24). This increases the susceptibility to EBV and HPV infections which subsequently progress into cancer (27–31). In addition, NKD compromises cytotoxicity against viruses during the infection allowing viral genes to be transfected into infected cells which transform into cancerous cells (23, 25). The arrows indicate the increased risk of developing conditions on the box that it is pointing to.

Most solid tumors observed in NKD patients are associated with oncogenic viral infections, especially Epstein Barr Virus (EBV), and Human Papilloma Virus (HPV). However, there are no reported cases of hepatitis B and C, HTLV-1, and KSHV induced cancers in NKD patients so far (23). The role of NK cells in virus driven malignancies is unclear although NK cells may indirectly suppress tumor growth as tumor cells have mechanisms of evading NK cell cytotoxicity (16). As with MDS-AML, NK activating receptors are downregulated in HPV-16 driven cervical cancer patients, indicating dysfunction of NK cells in these patients (24). The compromised NK cell cytotoxicity against oncogenic viral infection results in viral gene transfection (25) and inflammatory responses (26), both of which predispose to oncogenic changes, as observed in an Irish NKD patient with EBV-related lymphoma (20, 22) (Figure 1).

The effect of NKD on non-viral induced cancer remains unknown. A 56-year-old patient with FNKD developed multiple cutaneous squamous cell carcinoma (cSCC) unresponsive to chemoradiotherapy (32). However, her HPV serology was negative, hence cSCC was not induced by HPV (32). Similarly, NKD has been observed in a few patients with breast cancer and solid tumors but the mechanism of oncogenesis is unknown (33). Therefore, while NKD increases the risk of certain virus induced cancer, its effect on non-viral induced cancer not associated with GATA2 and MCM4 mutations is unknown.

The viral driven malignancies have been observed in patients with normal NK cells as well as in NKD patients (9, 33). While incidence of cancer in both groups cannot be compared due to small number of studies conducted across different periods of time among patients of various demographics, Spinner et al. (33) found that patients with normal NK cell counts were less likely to have major complications including severe infection and cancer than NKD patients. Also, such patients were likely to have fewer number of complications than those with low NK cell counts. Similarly, Imati et al. (9) reported higher relative risk of cancer incidence in patients with low natural cytotoxicity (1.0) in comparison to those with higher natural cytotoxicity (0.6). While the results were not specific to NK cells as natural cytotoxicity is exerted by several innate cells, the study illustrated the significance of natural cytotoxicity in increased incidence of cancer.

## NKD and Hodgkin's Lymphoma

The first case of viral-induced cancer in an NKD patient was observed in a 12-year-old girl with isolated CNKD who developed EBV related smooth muscle tumors (SMTs) in the bilateral adrenal glands (27). The patient had longstanding latent EBV infection, during which EBV transfects its gene into host DNA, facilitating development of cancer (27) (Figure 1). Therefore, the case suggests that NKD may have increased the risk of SMTs during latent EBV infection. Similar cases have been reported in a 6-year-old Taiwanese girl (28) and one of the three siblings (29, 30) with isolated NKDs who developed Hodgkin's lymphoma in the latent phase of EBV infection which may have been facilitated by NKD. While EBV downregulates NKG2D expression on NK cells to evade NK mediated cytotoxicity (23), NKD was present before primary EBV infection and persisted after complete recovery from the lymphoma in these patients (29, 30). Therefore, NKD was not transiently caused by EBV but it rather increased the risk of lymphoma (29, 30). In addition, NK cells have cytotoxicity against EBV infected B cells and they prevent transformation of EBV infected B cells into cancer in the acute EBV infection (23). Therefore, NKD increases the risk of EBV induced lymphoma by facilitating progression into cancer in acute and latent EBV infection. Furthermore, considering its antiviral immunity, NK cells may increase the risk of lymphoma by increasing the risk of developing EBV infection which subsequently progresses into lymphoma.

## NKD and Carcinoma *in situ* of Cervix

Finally, HPV driven cervical cancer is one of the most common virus driven malignancies in NKD patients with *GATA2* mutations. In a retrospective study of 57 patients with *GATA2* mutations, more than one third of patients had HPV driven malignancies (33). Similar to *GATA2* deficient patients with MDS-AML, patients with HPV driven malignancies had low number of CD56^bright^ NK cells (11). Spinner et al. found that almost all *GATA2* deficient patients with one or more complications of severe infections, MDS-AML, and pulmonary alveolar proteinosis had significantly decreased NK cell counts (33). This was in contrast to patients without such complications, whose median NK cell count fell within the normal range (33). This implied the crucial role of NK cells in preventing *GATA2* associated fatal diseases including HPV driven malignancies. Similarly, a 23-year-old girl diagnosed with recurrent carcinoma *in situ* of cervix (31) had peripheral blood mononuclear cells (PBMCs) which responded poorly against K562 on several occasions compared to the controls' PBMCs. The poor natural cytotoxicity was attributed to NK cell numbers, as the proportion of NK cells defined as CD3^−^CD16^+^ and CD3^−^NKH1^+^ cells was very low unlike CD3^+^ T cells. The NK cell selective markers were used to conclude that cytotoxicity of NKT and T cells was not sufficient to defense against HPV in the absence of NK cells. The observations imply the crucial role of NK cells on immunity against HPV driven malignancies (11).

## Conclusion

**Figure 2:**
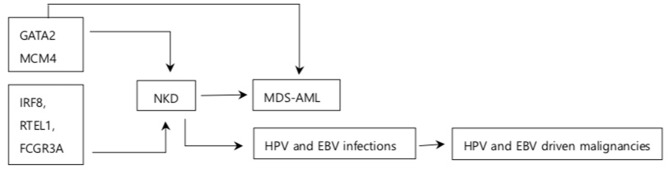
Summary of association between NKD and cancer. Arrows indicate association between the box it is emerging from and the box it is pointing to. GATA2, MCM4, IRF8, RTEL1, and FCGR3A are the genes responsible for NKD. GATA2 increases the risk of MDS-AML MCM4 is associated with increased NDA instability. NKD is associated with increased susceptibility to MDS-AML and some oncogenic viral infections. NKD has been observed in other solid tumors and breast cancer although association on between them is unknown.

In conclusion, most NKD cases with cancer have been observed in children and adolescents who have compromised immunity. The relatively small cohort size of NKD patients inevitably limits somewhat the generalization of the findings. Nevertheless, *GATA2* related autosomal dominant syndromes provide strong evidence that substitution and frameshift *GATA2* mutations at N-terminal to and within the second zinc finger cause both CNKD and MDS-AML. Both mice and human studies suggest that frameshift mutation at *MCM4* splice site results in the production of non-functional *MCM4* protein which increases susceptibility to DNA instability upon replication stress. *IRF8, RTEL1*, and *FCGR3A* mutations have been observed in NKD patients but their association with cancer is unknown. NKD may compromise anti-EBV immunity during acute and latent infections, facilitating the development of EBV induced cancer. However, the precise mechanisms of NK cell mediated EBV cytotoxicity need to be elucidated to consolidate such findings. The HPV induced cancer observed in CNKD patients imply the potential role of NK cells on HPV induced cancer. Therefore, NKD may increase the risk of cancers associated with certain viruses and mutations while future studies on mechanisms of cancer in NKD patients would highlight the role of NK cells in cancer immunity (Figure 2).

## Author Contributions

WM and SP: conception and design of the study, manuscript revision, and approval of the manuscript to be published. WM: literature review and drafting the manuscript.

### Conflict of Interest Statement

The authors declare that the research was conducted in the absence of any commercial or financial relationships that could be construed as a potential conflict of interest.
